# Supplementary material for: Pyroelectricity and field-induced spin-flop in (4-(Aminomethyl)pyridinium)2 MnCl4 · 2H2O
Source: R Soc Open Sci. 2020 May 27;7(5):200271. doi: 10.1098/rsos.200271 (PMC7277289; doi:10.1098/rsos.200271)
Supplement: Supplementary information [file rsos200271supp1.docx]

Pyroelectricity and field induced spin-flop in in (4-(Aminomethyl)pyridinium)_2_MnCl_4_·2H_2_O

Kaige Gao^1*^, Chunlin Liu^1^, Wei Zhang^1^, Kangni Wang^1^, Wenlong Liu^1^

^1^ College of Physical Science and Technology, Yangzhou University, Jiangsu 225009, P. R. China.





Fig. S1 The temperature dependent DSC of the compound **1**.





Fig. S2 Temperature dependence of inverse magnetic susceptibility, 1/χ, of compound 1 measured under magnetic field B = 5000 Oe.





Fig. S3 Derivative of the magnetization with respect to the field dM/dB for T = 2 K.
